# Aβ impairs nicotinic regulation of inhibitory synaptic transmission and interneuron excitability in prefrontal cortex

**DOI:** 10.1186/1750-1326-8-3

**Published:** 2013-01-17

**Authors:** Guo-Jun Chen, Zhe Xiong, Zhen Yan

**Affiliations:** 1 Department of Neurology, The First Affiliated Hospital of Chongqing Medical University, Chongqing 400016, China; 2 Department of Physiology and Biophysics, State University of New York at Buffalo, New York 14214, USA

**Keywords:** Alzheimer’s disease, β-amyloid, Nicotinic acetylcholine receptor, Prefrontal cortex, Interneuron, Pyramidal neuron, Inhibitory postsynaptic current, Firing, Protein kinase C

## Abstract

**Background:**

Accumulation of β-amyloid (Aβ) and cholinergic deficiency are two prominent features of Alzheimer’s disease (AD). To understand how Aβ-induced dysfunction of the nicotinic system may contribute to cognitive impairment in AD, we examined the effect of Aβ on nicotinic regulation of synaptic transmission and neuronal excitability in prefrontal cortex (PFC), a brain region critical for cognitive processes.

**Results:**

We found that activation of nicotinic acetylcholine receptors (nAChRs) with nicotine increased the inhibitory postsynaptic currents recorded in PFC pyramidal neurons, which was associated with the nicotine-induced increase in the excitability of PFC layer I GABAergic interneurons. Both effects of nicotine were disrupted by Aβ. However, Aβ did not impair nicotinic regulation of excitatory neurotransmission in PFC interneurons. The nicotinic effect on synaptic inhibition was also lost in transgenic mice with five familial Alzheimer’s disease mutations. Inhibiting PKC attenuated nicotinic regulation of inhibitory, but not excitatory, neurotransmission.

**Conclusions:**

Our study suggests that Aβ selectively impairs nicotinic regulation of inhibitory inputs to PFC pyramidal neurons, which might be due to its interference with PKC activation. Thus, in the PFC circuits of AD, the balance between inhibition and excitation under the control of nAChRs may be disturbed by Aβ.

## Background

Aβ plays a critical role in the pathogenesis of Alzheimer’s disease (AD) [1-4]. Earlier studies show that Aβ can cause cell death in cultured neurons [5] by inducing oxidative stress and disrupting intracellular calcium homeostasis [6]. Recent evidence suggests that the cognitive deficit and memory loss in early stages of AD are due to synaptic failure [7] and functional changes in the activity of neuronal network [8]. However, the role of Aβ in synaptic dysfunction in AD is far from clear.

In addition to Aβ accumulation, another hallmark of AD is a selective loss of cholinergic neurons in basal forebrain [9], and the loss of nicotinic acetylcholine receptors (nAChRs) in the hippocampus and cortex [10,11]. Neuronal nAChR is a pentameric structure formed from α- and β- subunits (α2-α10, β2-β4). nAChRs are distributed in both presynaptic and postsynaptic sites [12,13], regulating transmitter release, synaptic response and neuronal excitability [14]. Animal studies have found that nAChR activation improves working memory [15,16], while nAChR antagonist, mecamylamine, impairs attention accuracy or reaction time [17,18]. Clinical drugs targeting nAChRs improve symptoms in AD patients [19].

It has been suggested that nAChRs are involved in regulating the integrated circuit activity between interneurons and projection neurons [20,21]. We hypothesize that Aβ may interrupt nicotinic regulation of inhibitory/excitatory balance at cortical circuits, thus contributing to the loss of cognition and memory in AD. To test this, we examined the effect of nicotine on inhibitory inputs to cortical pyramidal neurons and the excitatory inputs to cortical interneurons, as well as the potential impact of Aβ on the regulatory effects of nAChRs.

## Results

### Nicotine increases inhibitory neurotransmission in PFC layer V pyramidal neurons through non-α7 nACh receptors

**Figure 1:**
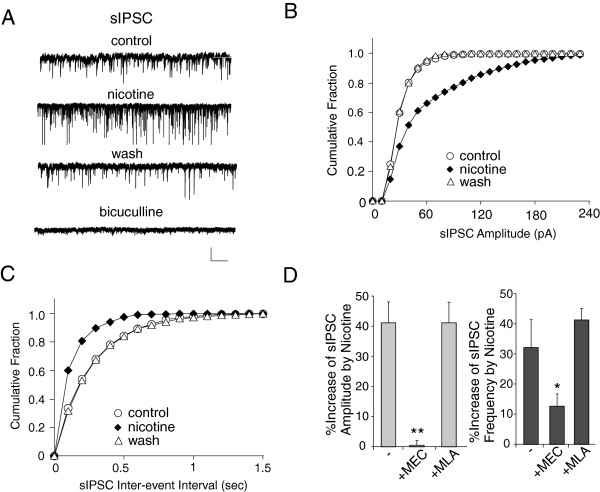
**Nicotine enhanced sIPSC in PFC layer V pyramidal neurons. A-D,** Representative sIPSC traces (**A**) and cumulative plots of the distribution of sIPSC amplitude (**B**) and frequency (**C**) in the absence (control, wash) or presence of nicotine (5 μM) recorded in a PFC pyramidal neuron. The blockade of sIPSC by bicuculline (10 μM) was also shown in A. Scale bars (**A**): 50 pA, 5s. **D,** Bar plot summary of the percentage increase of sIPSC amplitude and frequency by nicotine in the absence or presence of MEC (mecamylamine, 10 μM, a non-α7 nAChR antagonist) or MLA (methyllycaconitine, 1 μM, an α7 nAChR antagonist). *: p < 0.05, **: p < 0.005, ANOVA.

We first assessed the effect of nAChR activation on inhibitory neurotransmission in layer V pyramidal neurons in the prefrontal cortex (PFC). Nicotine (5 μM, [22,23]) was applied to the bath solution for 10 min. As shown in Figure 1A-D, nicotine significantly increased sIPSC amplitudes, as indicated by a rightward shift of their distribution. Moreover, nicotine significantly increased sIPSC frequencies, as indicated by a leftward shift of the distribution of inter-event intervals. The effect of nitonine on sIPSC was reversible. Note that sIPSC was completely blocked by bicuculline (10 μM), indicating its mediation by GABA_A_ receptors (Figure 1A). In the sample of cells we tested, nicotine increased sIPSC amplitude by 41.1 ± 7.0% and frequency by 32.1 ± 9.3% (n = 9, Figure 1D).

To identify the possible receptor subtypes that mediate the nicotinic effect, we applied nAChR antagonists to PFC slices. As shown in Figure 1D, mecamylamine (MEC, 10 μM), a non-α7 nAChR antagonist, largely blocked the effect of nicotine on sIPSC amplitude (0.4 ± 1.6%, n = 5) and frequency (12.6 ± 4.1%, n = 5). In contrast, in the presence of methyllycaconitine (MLA, 1 μM), an α7 nAChR antagonist, the effect of nicotinic on sIPSC was not significantly changed (amplitude: 41.1 ± 6.9%, frequency: 41.3 ± 3.8%, n = 6). It suggests that the nicotinic effect on sIPSC in layer V PFC pyramidal neurons is mediated by non-α7 nAChRs.

### Nicotine boosts the excitability of PFC layer I GABAergic interneurons

To assess whether nicotinic enhancement of sIPSC is due to nAChR-induced increase in the excitability of PFC GABAergic interneurons, we examined the effect of nicotine on action potential firing of interneurons in PFC. Since layer I of neocortex contains exclusively GABAergic interneurons with extensive axonal plexus innervating neurons in deep layers [24-26], we focused our studies on PFC layer I GABAergic interneurons.

**Figure 2:**
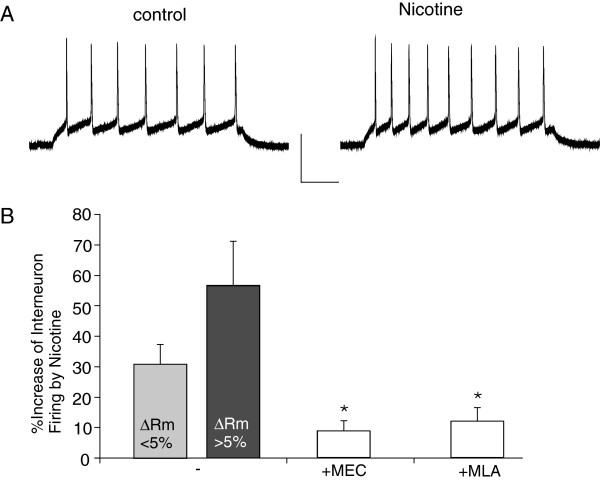
**Nicotine increased the excitability of PFC layer I GABAergic interneurons. A,** Representative spikes elicited by a depolarizing current pulse in the absence (control) or presence of nicotine (5 μM) recorded in a PFC layer I GABAergic interneuron. Scale bars: 50mV, 100ms. **B,** Bar plot summary of the percentage increase of firing rate by nicotine in layer I interneurons in the absence or presence the antagonist MEC (10 μM) or MLA (1 μM). Note that the effect of nicotine was calculated in two different groups based on the change of resting potential (ΔRm) by nicotine. *: p < 0.05, ANOVA.

As shown in Figure 2A, nicotine caused a significant increase in the spike number in the representative layer I interneuron. We noticed that nicotine caused a significant change in the resting potential in a portion of layer I interneurons (6/15, ΔRm >5%, from −66.4 ± 3.5 mV to −60.4 ± 3.4 mV), but not in others (9/15, ΔRm <5%, from −68.7 ± 2.9 mV to −67.3 ± 3.0 mV). The Rm change is similar to the nicotine-induced depolarization in some hippocampal neurons [27]. So we divided these cells into two groups before evaluating the effect of nicotine on the firing rate. As shown in Figure 2B, nicotine significantly increased the firing rate in both groups (ΔRm < 5%: 30.8 ± 6.7%, n = 9; ΔRm > 5%: 56.5 ± 16.4%, n = 6). In the presence of nAChR antagonist MEC or MLA, nicotine failed to change the resting potential in all neurons we tested (MEC-treated cells: -60.9 ± 2.2 mV to −60.4 ± 2.3 mV, n = 7; MLA-treated cells: -70.7 ± 1.8 to −69.7 ± 1.8 mV, n = 8). However, the nicotinic effect on firing rate was significantly attenuated by MEC (Figure 2B, 8.6 ± 3.3%, n = 7) or MLA (Figure 2B, 11.8 ± 4.4%, n = 8), suggesting that both non-α7 and α7 nAChRs are involved in nicotinic enhancement of interneuron excitability.

We also assessed whether nicotine affects the excitability of deep layer neurons. We found that nicotine did not cause a significant increase in the firing rate or resting potential of layer V fast-spiking interneurons (n = 5). Nicotine also failed to show any significant effect on the firing rate of layer V pyramidal neurons (n = 5).

### Aβ attenuates the nicotinic effect on inhibitory transmission in layer V cortical pyramidal neurons

**Figure 3:**
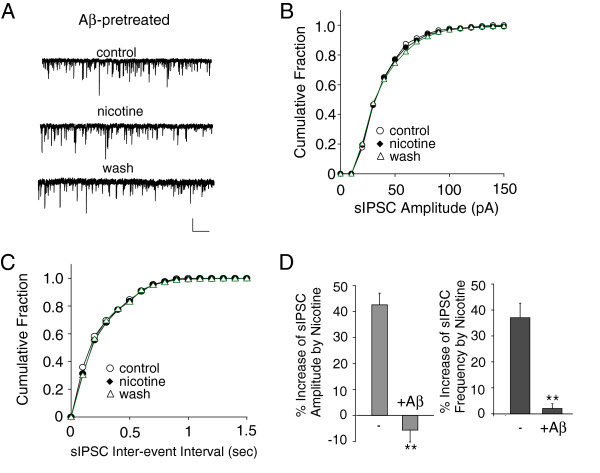
**Aβ diminished the nicotinic effect on sIPSC in PFC layer V pyramidal neurons. A-C,** Representative sIPSC traces (**A**) and cumulative plots of the distribution of sIPSC amplitude (**B**) and frequency (**C)** in the absence (control) or presence of nicotine (5 μM) recorded in a PFC pyramidal neuron pre-incubated with Aβ oligomer (1 μM, 3 hours). Scale bars **(A**): 100pA, 15s. **D,** Bar plot summary of the percentage change of sIPSC amplitude and frequency by nicotine in PFC pyramidal neurons with or without Aβ pretreatment. *: p < 0.05, **: p < 0.01, *t*-test.

Given the involvement of cholinergic hypofunction in AD, we next examined whether Aβ could alter the nicotinic regulation of inhibitory synaptic transmission in PFC pyramidal neurons. Aβ oligomers were generated as we described and characterized before [28]. PFC slices were incubated with Aβ oligomers (1 μM, [28-30]) for at least 3 hours before recording. No significant difference in basal sIPSC was observed after Aβ treatment (non-treated: 35.2 ± 2.2 pA, 5.5 ± 1.1 Hz, n = 5; Aβ-treated: 36.8 ± 2.4 pA, 6.1 ± 1.0 Hz, n = 5). However, nicotine failed to increase the amplitude or frequency of sIPSC in Aβ-treated neurons. A representative example is shown in Figure 3A-C. As summarized in Figure 3D, in layer V pyramidal neurons pretreated with Aβ, the nicotine-induced change in sIPSC amplitude (−5.6 ± 4.47%, n = 5) and frequency (2.03 ± 1.87%, n = 5) was significantly smaller than that in non-treated control cells (amplitude: 41.2 ± 5.1%, frequency: 36.4 ± 7.8%, n = 4).

**Figure 4:**
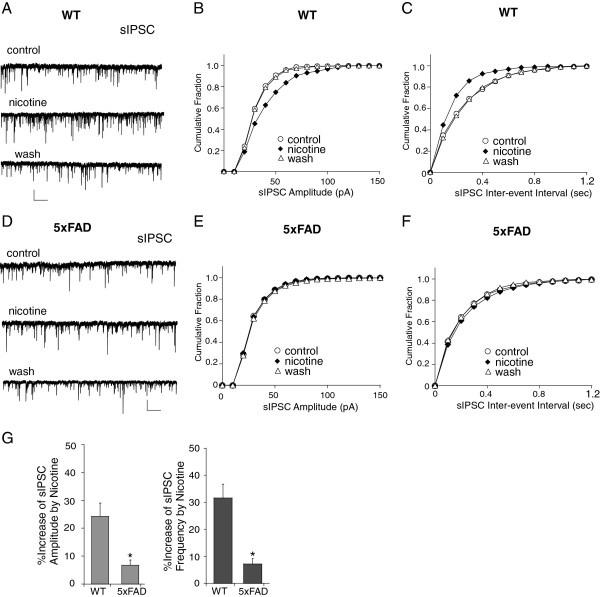
**The nicotinic effect on sIPSC was lost in PFC pyramidal neurons from 5xFAD mice. A-F,** Representative sIPSC traces (**A, D**) and cumulative plots of the distribution of sIPSC amplitude (**B, D**) and frequency (**C, E**) in the absence (control, wash) or presence of nicotine (5 μM) recorded in layer V PFC pyramidal neurons from WT vs. 5xFAD mice. Scale bars (**A**): 50 pA, 5 s. ***G*,** Bar plot summary of the percentage increase of sIPSC amplitude and frequency by nicotine in WT vs. 5xFAD mice. *: p < 0.05, *t*-test.

To confirm that the observed effect of Aβ also occurs *in vivo*, we examined the transgenic mice with five familial Alzheimer’s disease mutations (5xFAD), which rapidly recapitulate major features of AD amyloid pathology. These mice start to accumulate intraneuronal Aβ42 at ~1.5 months of age within neuron soma and neurites and amyloid deposition reaches a very large burden in deep cortical layers [31]. As shown in Figure 4, nicotine (5 μM) had almost no effect on sIPSC in 1.2-month-old 5xFAD mice (amplitude: 6.7 ± 1.8%, frequency: 7.1 ± 1.9%, n = 4), which was significantly different from the enhancing effect of nicotine on sIPSC in age-matched WT mice (amplitude: 24.2 ± 4.8%, frequency: 31.6 ± 5.2%, n = 6). These results suggest that Aβ impairs nicotinic regulation of inhibitory synaptic transmission in PFC.

### Aβ weakens the nicotinic effect on the excitability of PFC layer I GABAergic interneurons

**Figure 5:**
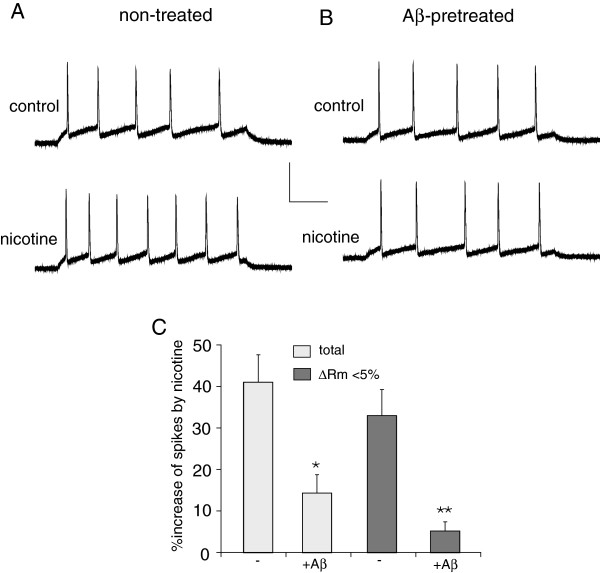
**Aβ attenuated the nicotinic effect on layer I interneuron excitability. A, B,** Representative spikes elicited by a depolarizing current pulse in the absence (control) or presence of nicotine (5 μM) recorded in PFC layer I GABAergic interneurons without (**A**) or with Aβ pre-treatment (**B**). Scale bars: 50mV, 100ms. **C,** Bar plot summary of the percentage increase of firing rate by nicotine in non-treated (−) and Aβ-pretreated layer I interneurons. Note that the effect of nicotine was calculated in two different groups based on the change of resting potential (ΔRm) by nicotine. *: p < 0.05, **: p < 0.01, ANOVA.

Since the nicotinic effect on sIPSC is due to its regulation of the excitability of GABAergic interneurons, we next examined whether Aβ also interferes with the nicotinic effect on action potential firing of layer I interneurons. PFC slices were pretreated with Aβ oligomers (1 μM) for at least 3 hours before recording. Nicotine significantly increased the firing rate in non-treated control neurons, but not in Aβ-treated neurons. Representative examples are shown in Figure 5A and 5B. As summarized in Figure 5C, in all the neurons pooled together (ΔRm > 5% and ΔRm < 5%), nicotine caused a significantly smaller effect on the firing rate of Aβ-treated interneurons (14.3 ± 8.5%, n = 9) than control cells (41.1 ± 7.5%, n = 15). In the subgroups with ΔRm < 5%, the effect of nicotine on the firing rate in Aβ-treated cells (5.2 ± 2.4%, n = 7) was also significantly smaller than that in control cells (33.0 ± 6.2%, n = 9). It suggests that Aβ impairs nicotinic regulation of GABAergic interneuron excitability in PFC.

### Aβ does not interfere with nicotinic regulation of excitatory neurotransmission

**Figure 6:**
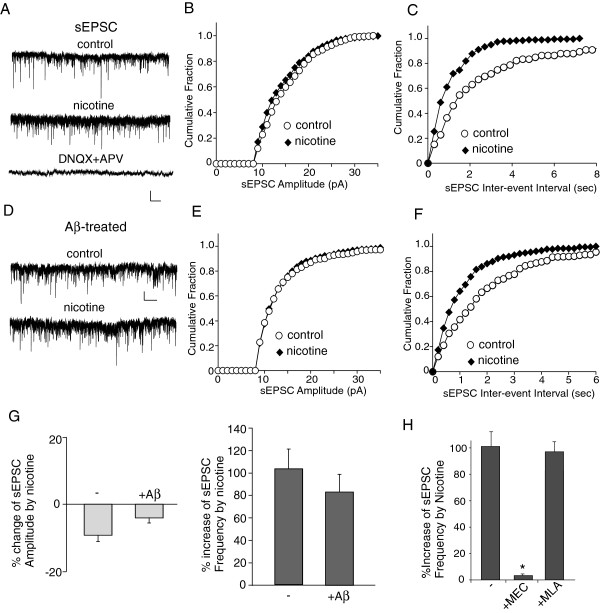
**Aβ did not alter the nicotinic effect on sEPSC in layer I interneurons. A-C,** Representative sEPSC traces (**A**) and cumulative plots of the distribution of sEPSC amplitude (**B**) and frequency (**C**) in the absence (control) or presence of nicotine (5 μM) recorded in a PFC layer I interneuron. The blockade of sEPSC by DNQX (20 μM) and APV (40 μM) was also shown in A. Scale bars (**A**): 10pA, 15s. ***D*-*F*,** Representative sEPSC traces (**D**) and cumulative plots of the distribution of sEPSC amplitude (**E**) and frequency (**F**) in the absence or presence of nicotine recorded in a PFC layer I interneuron pre-treated with Aβ (1 μM, 3 hours). Scale bars (**D**): 10pA, 15s. **G,** Bar plot summary of the percentage change of sEPSC amplitude and frequency by nicotine in non-treated (−) and Aβ-pretreated layer I interneurons. **H,** Bar plot summary of the percentage increase of sEPSC frequency by nicotine in the absence or presence of MEC (10 μM) or MLA (1 μM). *: p < 0.005, ANOVA.

Given the Aβ-induced impairment of nicotinic regulation of inhibitory transmission and interneuron excitability, we would like to know whether Aβ also impairs nicotinic regulation of excitatory transmission. To do so, we examined the effect of nicotine on spontaneous EPSC (sEPSC) in layer I interneurons treated with or without Aβ. As shown in Figure 6A-C, nicotine caused a significant increase in the sEPSC frequency, but not sEPSC amplitude, in non-treated control cells. Similar effects were observed in Aβ-treated cells (Figure 6D-F). Note that sEPSC was completely blocked by DNQX (20 μM) plus APV (40 μM), indicating its mediation by glutamate receptors (Figure 6A). As summarized in Figure 6G, the nicotine-induced enhancement of sEPSC frequency was not significantly altered by Aβ treatment (non-treated: 103.3 ± 27.5%, n = 10; Aβ-treated: 83.0 ± 25.9%, n = 9).

Why does Aβ differentially affect nicotinic regulation of excitatory vs. inhibitory neurotransmission? One possibility is that distinct nAChR subtypes mediate the nicotinic effect on sEPSC or sIPSC. To test this, we applied specific nAChR antagonists. As shown in Figure 6H, in cells treated with the non-α7 nAChR antagonist MEC (10 μM), nicotine failed to increase sEPSC frequency (2.6 ± 1.6%, n = 4). In contrast, the nicotinic enhancement of sEPSC frequency (97.9 ± 9.9%, n = 4) was intact in cells treated with the α7 nAChR antagonist MLA (1 μM). It suggests that non-α7 nAChRs play a major role in mediating the nicotinic effect on excitatory neurotransmission, which is similar to their major role in mediating the nicotinic effect on inhibitory neurotransmission. Thus, the differential effects of Aβ on nicotinic regulation of sIPSC and sEPSC can not be explained by the nAChRs involved.

### PKC is differentially involved in nicotinic regulation of inhibitory vs. excitatory neurotransmission

Having excluded the possibility that nAChR subtypes may contribute to the selective effect of Aβ on nicotinic regulation of inhibitory transmission, we hypothesize that Aβ might interfere with some downstream signaling molecule that is differentially involved in the nicotinic regulation of sIPSC or sEPSC. Our previous studies have found that PKC activation is often impaired by Aβ treatment [30,32,33], so we examined the involvement of PKC in nicotinic regulation of sIPSC and sEPSC. PFC slices were pre-incubated with a PKC inhibitor for at least 1 hour, and sIPSC was recorded in layer V pyramidal neurons and sEPSC was recorded in layer I GABAergic interneurons.

**Figure 7:**
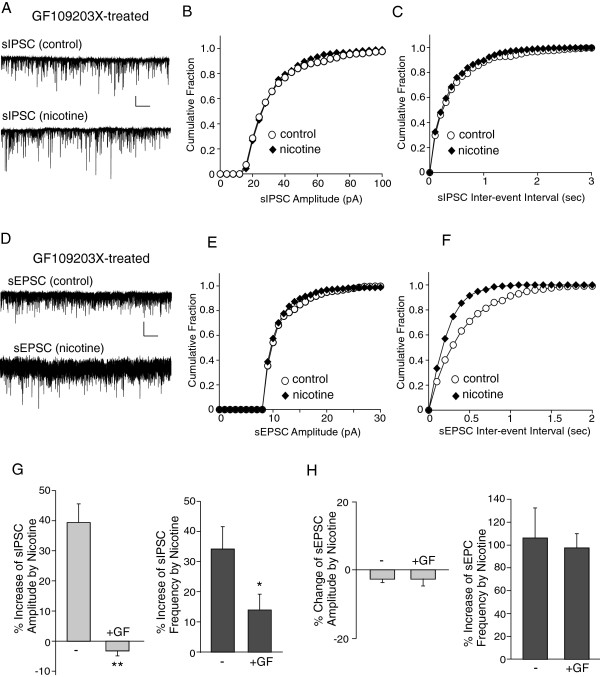
**Inhibiting PKC blocked the nicotinic effect on sIPSC but not sEPSC. A-C,** Representative sIPSC recordings (**A**) and cumulative plots of the distribution of sIPSC amplitude (**B**) and frequency (**C**) in the absence (control) or presence of nicotine (5 μM) recorded in a PFC pyramidal neuron pretreated with the PKC inhibitor GF109203X (1 μM). Scale bars (**A**): 100pA, 15s. **D-F,** Representative sEPSC traces (**D**) and cumulative plots of the distribution of sEPSC amplitude (**E**) and frequency (**F**) in the absence (control) or presence of nicotine (5 μM) recorded in a PFC layer I interneuron pretreated with GF109203X (1 μM). Scale bars (**D**): 10pA, 15s. **G, H,** Bar plot summary of the percentage change of sIPSC (**G**) or sEPSC (**H**) amplitude and frequency by nicotine in neurons pretreated without (−) or with GF109203X. *: p < 0.05, **: p < 0.01, *t*-test.

As shown in Figure 7A-C, in PFC pyramidal neurons treated with the potent and selective PKC inhibitor GF109203X (1 μM), nicotine failed to cause a significant increase in the sIPSC amplitude (−3.2 ± 1.7%, n = 7, Figure 7G), and had a significantly attenuated effect on sIPSC frequency (18.9 ± 8.7%, n = 7), which was contrary to the effects of nicotine on sIPSC in control cells (sIPSC amplitude: 39.4 ± 1.7%; sIPSC frequency: 34.2 ± 7.4%, n = 5). However, nicotine still caused a significant increase in the sEPSC frequency in GF109203X-treated interneurons (Figure 7D-F, 97.4 ± 12.6%, n = 8, Figure 7H), similar to its effect on sEPSC frequency in control cells (106.1 ± 26.3%, n = 5).

**Figure 8:**
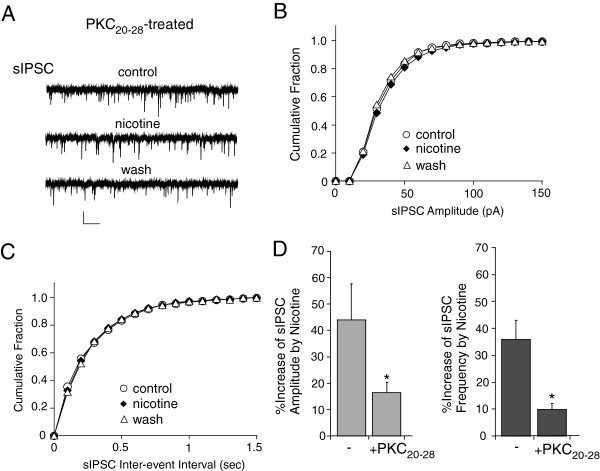
**Highly specific PKC inhibitor blocked the nicotinic effect on sIPSC. A-C,** Representative sIPSC traces (**A**) and cumulative plots of the distribution of sIPSC amplitude (**B**) and frequency (**C**) in the absence (control) or presence of nicotine (5 μM) recorded in a PFC pyramidal neuron pretreated with myristolated PKC inhibitor 20–28 (10 μM). Scale bars (**A**): 50 pA, 5 s. **D,** Bar plot summary of the percentage increase of sIPSC amplitude and frequency by nicotine in neurons pretreated without (−) or with myr-PKC_20-28_. *: p < 0.05, *t*-test.

To further confirm the involvement of PKC in nicotinic regulation of synaptic inhibition, we also treated neurons with a different type of highly specific PKC inhibitor, PKC_20-28_, which is a peptide containing the pseudosubstrate sequence from PKC α and PKC β. N-Terminus is myristoylated to allow membrane permeability. As shown in Figure 8, in myr-PKC_20-28_ (10 μM, 1 hr)-treated neurons, nicotine (5 μM) had significantly diminished enhancing effect on mIPSC amplitude (control: 44.1 ± 13.6%, n = 4, PKC_20-28_: 16.5 ± 3.8%, n = 5) and frequency (control: 36.0 ± 7.1%, n = 4, PKC_20-28_: 9.9 ± 2.2%, n = 5). These results suggest that PKC activation is required for nicotinic regulation of inhibitory, but not excitatory, synaptic transmission in PFC.

## Discussion

Our experiments have shown that, in rat prefrontal cortex, 1) nicotine increased sIPSC in layer V pyramidal neurons and the firing rate in layer I interneurons, both of which were disrupted by Aβ; 2) Aβ did not impair nicotinic regulation of sEPSC in layer I interneurons; 3) PKC inhibitors differentially interfered with nicotinic regulation of excitatory or inhibitory transmission, mimicking the effect of Aβ. These results suggest that Aβ selectively impairs nicotinic regulation of inhibitory inputs to cortical pyramidal neurons, which may involve a PKC-dependent mechanism.

Nicotine often has neuron-specific effects in different networks, which are mediated by different nAChR subtypes [21,34,35]. In this study, we have found that nicotine enhances both inhibitory inputs to PFC layer V pyramidal neurons and excitatory inputs to layer I interneurons. In the presence of non-α7 nAChR antagonist MEC, both sIPSC and sEPSC are irresponsive to nicotine, suggesting that α4β2 may mediate these effects of nicotine [20,36]. Nicotine does not alter the excitability of layer V pyramidal neurons, but significantly increases the firing rate of layer I interneurons, an effect mediated by both α7 and non-α7 nAChRs. Involvement of different nAChRs may also suggest distinct roles of nicotine in regulating neuronal functions. In hippocampal cultures, α7 and α4β2 displayed distinct patterns of expression, with α7 preferentially present on the somatodendrites whereas α4β2 distributed on both the axonal and dendritic compartments [37]. Our results are consistent with the finding that presynaptic α4β2 receptors contribute to neurotransmission [38,39] and both somatodendritic α7 and presynaptic α4β2 receptors modulate neuronal excitability [12,40,41].

Both Aβ accumulation and nicotinic deficits occur in the progression of AD [42,43]. Aβ peptide may alter nicotinic function in several ways. Direct binding of Aβ_1–42_ to α7 receptors leads to inhibition of channel open probability [44] and ionic current [43,45]. However, several lines of evidence suggest that direct inhibition of nAChRs by Aβ might not be enough to explain Aβ-induced impairment of nicotinic functions. In hippocampal interneurons of transgenic mice overexpressing Aβ, α7 nAChRs are still functioning [46]. Aβ is able to elevate presynaptic calcium levels, which could occlude the enhancing effect of nicotine on calcium-dependent transmitter release [47]. Calcium and PKC have been found to be involved in nicotine-facilitated neurotransmission in interneurons [48,49]. Our results demonstrate that PKC inhibitor has a similar effect on nicotinic regulation of synaptic transmission as Aβ, suggesting that PKC could be an important mediator in Aβ-induced impairment of nAChR functions in AD. It supports the idea that PKC-related intervention might be promising for AD treatment [50-52].

In this study, we have found that nicotinic regulation of interneuron firing and GABAergic inputs to pyramidal neurons are selectively susceptible to Aβ, while nicotinic regulation of excitatory inputs to interneurons is relatively preserved. It suggests that interneuron-mediated inhibition and its excitability are Aβ targets. It is known that inhibitory terminals of fast-spiking interneurons are better equipped to support prolonged transmitter release at a higher frequency in comparison with pyramidal neurons [53]. Electrical and chemical connections of cortical interneurons promote their synchronous firing, thus interneurons play an important role in coordinating cortical activity [54,55], which is critical for working memory [56]. The Aβ-induced selective impairment of nicotinic regulation of inhibitory inputs to cortical principal neurons could contribute to the dysfunction of neuronal network and the imbalance of inhibition/excitation, leading to interruption of working memory.

## Methods

### Drugs

Aβ oligomer was prepared as described previously [28,57]. Nicotine (Sigma), mecamylamine (Sigma), methyllycaconitine (Tocris), GF109203X (Tocris), myr-PKC_20-28_ (Calbiochem) were made freshly from stocks.

### Animals

The transgenic mice that coexpress a total of five FAD mutations [APP K670N/M671L (Swedish) + I716V (Florida) + V717I (London) and PS1 M146L + L286V] [31] was a generous gift from Dr. William E. Van Nostrand (Stony Brook University). Transgenic males (Hets) were bred with mature females (WT). Genotyping were performed by PCR.

### Slice preparation

All experiments were performed under the supervision of State University of New York at Buffalo Animal Care Committee. Sprague Dawley rats (3–5 weeks old) were anesthetized with 2-bromo-2-chloro-1,1,1-trifluoroethane (1 ml/100 g) inhalation before decapitation [28,58]. Brains were quickly removed and sliced (300 μm) with a Leica (Nussloch, Germany) VP1000S Vibratome. Slices were then incubated in artificial CSF (in mM: 130 NaCl, 26 NaHCO_3_, 3 KCl, 5 MgCl_2_, 1.25 NaH_2_PO_4_, 2 CaCl_2_, and 10 glucose, pH 7.4, 300 mOsm) bubbled with 95% O_2_ and 5% CO_2_.

### Patch clamp recordings

Voltage clamp recording of synaptic currents in slices was performed as described previously [33,59]. The PFC slice was placed in a perfusion chamber attached to the fixed stage of an upright microscope (Olympus Optical, Melville, NY) and submerged in continuously flowing ACSF. For sIPSC recording, patch electrode (3–5 MΩ) was filled with the following solution (in mM): 100 CsCl, 10 HEPES, 1 MgCl_2_, 1 EGTA, 30 *N*-methyl-d-glucamine (NMG), 5 MgATP, 0.5 Na_2_GTP and 12 phosphocreatine, pH 7.2–7.3, 270–280 mOsm. CNQX or DNQX (20 μM) and APV (40 μM) were added to ACSF to block AMPA and NMDA receptors. For sEPSC recording, the internal solution was composed of (in mM): 130 Cs-methanesulfonate, 10 HEPES, 10 CsCl, 4 NaCl, 1 MgCl_2_, 1 EGTA, 5 NMG, 5 MgATP, and 0.5 Na_2_GTP and 12 phosphocreatine, pH 7.2, 275–290 mOsm. Bicuculline (10 μM) was added to ACSF to block GABA_A_ receptors. Cells were visualized with a 40x water-immersion lens and illuminated with near infrared (IR) light, and the image was detected with an IR-sensitive CCD camera. A Multiclamp 700A amplifier (Axon Instruments) was used for the recording. Tight seals (2–10 GΩ) from visualized neurons were obtained by applying negative pressure. The membrane was disrupted with additional suction, and the whole-cell configuration was obtained. The access resistances were 13–18 MΩ. The membrane potential was held at −70 mV.

Mini Analysis program (Synaptosoft, Leonia, NJ) was used to analyze synaptic activity. Individual synaptic events with fast onset and exponential decay kinetics were captured with threshold detectors in Mini Analysis software. All quantitative measurements were taken 4–6 min after drug application. Spontaneous IPSC or EPSC recordings of 3 min under each condition were used for obtaining cumulative distribution plots of the amplitudes and inter-event intervals. To measure cell excitability, the whole-cell current-clamp technique [33,59] was used to record spikes evoked by a 500ms depolarizing current pulse. The amplitude of injected current was adjusted so that 5–7 spikes were elicited in the control ACSF solution. The patch electrode was filled with an internal solution containing (in mM): 60 K_2_SO_4_, 60 NMG, 40 HEPES, 4 MgCl_2_, 0.5 BAPTA, 12 phosphocreatine, 2 Na_2_ATP and 0.2 Na_3_GTP, pH 7.2–7.3, 265–270 mOsm. The firing rate of each neuron was averaged from 10 consecutive traces under each condition.

### Data analysis

Statistical comparisons of the synaptic currents were made using the Kolmogorov-Smirnov (K-S) test. Experiments with two groups were analyzed statistically using unpaired Student’s *t*-tests. Experiments with more than two groups were subjected to one-way ANOVA, followed by *post hoc* Tukey tests.

Numerical values were expressed as mean ± SEM.

## Competing interests

The authors declare that they have no competing interests.

## Authors’ contributions

GJC and ZX performed experiments and analyzed data. ZY designed experiments and wrote the manuscript. All authors read and approved the final manuscript.
